# Pseudogap phase of cuprate superconductors confined by Fermi surface topology

**DOI:** 10.1038/s41467-017-02122-x

**Published:** 2017-12-11

**Authors:** N. Doiron-Leyraud, O. Cyr-Choinière, S. Badoux, A. Ataei, C. Collignon, A. Gourgout, S. Dufour-Beauséjour, F. F. Tafti, F. Laliberté, M.-E. Boulanger, M. Matusiak, D. Graf, M. Kim, J.-S. Zhou, N. Momono, T. Kurosawa, H. Takagi, Louis Taillefer

**Affiliations:** 1 Institut Quantique, Département de Physique & RQMP, Université de Sherbrooke, Sherbrooke, QC J1K 2R1 Canada; 2 Institute of Low Temperature and Structure Research, Polish Academy of Sciences, 50-422 Wrocław, Poland; 3 National High Magnetic Field Laboratory, Florida State University, Tallahassee, FL 32306 USA; 4 École Polytechnique, CNRS, Université Paris-Saclay, 91128 Palaiseau, France; 5 Collège de France, 75005 Paris, France; 6 Materials Science and Engineering Program/Mechanical Engineering, University of Texas- Austin, Austin, TX 78712 USA; 7 Department of Applied Sciences, Muroran Institute of Technology, Muroran, 050-8585 Japan; 8 Department of Physics, Hokkaido University, Sapporo, 060-0810 Japan; 9 Department of Advanced Materials, University of Tokyo, Kashiwa, 277-8561 Japan; 10 Canadian Institute for Advanced Research, Toronto, ON M5G 1Z8 Canada

## Abstract

The properties of cuprate high-temperature superconductors are largely shaped by competing phases whose nature is often a mystery. Chiefly among them is the pseudogap phase, which sets in at a doping p* that is material-dependent. What determines p* is currently an open question. Here we show that the pseudogap cannot open on an electron-like Fermi surface, and can only exist below the doping *p*_FS_ at which the large Fermi surface goes from hole-like to electron-like, so that p* ≤ *p*_FS_. We derive this result from high-magnetic-field transport measurements in La_1.6−*x*_Nd_0.4_Sr_*x*_CuO_4_ under pressure, which reveal a large and unexpected shift of p* with pressure, driven by a corresponding shift in *p*_FS_. This necessary condition for pseudogap formation, imposed by details of the Fermi surface, is a strong constraint for theories of the pseudogap phase. Our finding that p* can be tuned with a modest pressure opens a new route for experimental studies of the pseudogap.

## Abstract (web summary)

High-temperature superconductors exhibit pseudogap behaviour that remains of unknown origin, despite many years of intensive study. Here the authors study the onset of the pseudogap under pressure, providing evidence that it requires a hole-like Fermi surface and constraining future theoretical developments.

## Introduction

A central puzzle of cuprate superconductors^1^, the pseudogap is a partial gap that opens in their spectral function, detected most directly by angle-resolved photoemission spectroscopy (ARPES). It opens below a temperature T* that decreases monotonically with increasing hole concentration (doping) *p*. For example, T* = 130 ± 20 K in La_2-*x*_Sr_*x*_CuO_4_ (LSCO) at *p* = 0.15^2^ and T* = 75 ± 5 K in La_1.6−*x*_Nd_0.4_Sr_*x*_CuO_4_ (Nd-LSCO) at *p* = 0.20^3^. Transport properties like the electrical resistivity ρ(*T*) and the Nernst coefficient ν(*T*) are affected by the opening of the pseudogap and so may be used to detect T*, as previously reported in refs. ^4,5^ and ref. ^6^, respectively.

**Fig. 1:**
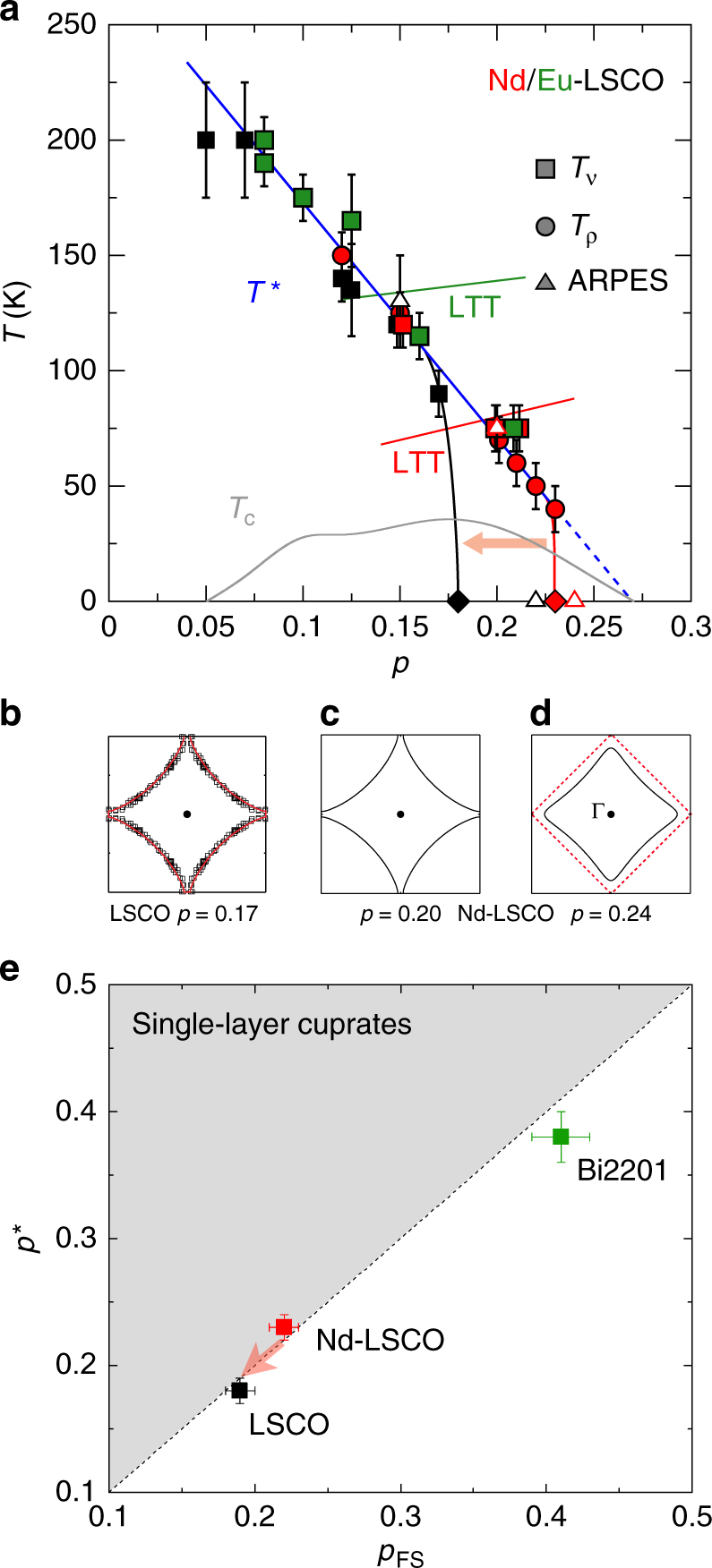
Phase diagram and correlation between p* and *p*_FS_. **a** Temperature-doping phase diagram, showing the pseudogap temperature T* measured by ARPES (triangles), electrical resistivity (circles) and Nernst effect (squares), in LSCO (black), Nd-LSCO (red) and Eu-LSCO (green) (adapted from ref. ^6^; see references therein). The diamonds mark the critical doping p* at which the pseudogap phase ends at *T* = 0 (in the absence of superconductivity), for LSCO (black; p* = 0.18, refs. ^8,9^) and Nd-LSCO (red; p* = 0.23, refs. ^4,5^). The short solid lines mark the transition into the low-temperature tetragonal structure (LTT) for Eu-LSCO (green; ref. ^36^ and Nd-LSCO (red; ref. ^34^), in the interval where it crosses T*. The grey line is the superconducting critical temperature *T*_c_ of LSCO. The dashed line is a linear extension of the T* line (blue). The red arrow illustrates the effect of applying hydrostatic pressure to Nd-LSCO: it shifts p* down in doping. **b**,** c**,** d** Sketch of the Fermi surface in the first Brillouin zone (frame), as measured by ARPES in LSCO (ref. ^11^) at *p* = 0.17 (**b**) and in Nd-LSCO (ref. ^3^) at *p = *0.20 (**c**) and *p = *0.24 (**d**). The red dashed line is the anti ferromagnetic zone boundary (AFZB), which intersects the hole-like Fermi surface of Nd-LSCO at *p = *0.20, but not its electron-like surface at *p = *0.24. **e** Correlation between p* and *p*_FS_ in single-layer cuprates. p* is measured in the normal state at *T = *0, by high-field transport in Nd-LSCO (p* = 0.23 ± 0.01, ref. ^5^) and LSCO (p* = 0.18 ± 0.01, refs. ^8,9^), and by high-field NMR in Bi2201 (p* = 0.38 ± 0.02, ref. ^10^). *p*_FS_ is measured by ARPES in LSCO (*p*_FS_ = 0.19 ± 0.01, refs. ^2,11^), Nd-LSCO (*p*_FS_ = 0.22 ± 0.01; ref. ^3^) and Bi2201 (*p*_FS_ = 0.41 ± 0.02, ref. ^12^). The red arrow shows the effect of applying pressure to Nd-LSCO (red square). We find that p* and *p*_FS_ decrease in tandem, preserving the equality p* = *p*_FS_ and thereby showing that p* is constrained by the condition p* ≤ *p*_FS_. The grey shading marks the forbidden region

In Fig. 1a, we show the temperature-doping phase diagram of LSCO, Nd-LSCO and La_1.8-*x*_Eu_0.2_Sr_*x*_CuO_4_ (Eu-LSCO). We see that all three materials have the same T* up to *p* ~0.17, irrespective of their different crystal structures^6^. Indeed, Nernst data^6^ show, for example, that T* = 120 ± 10 K at *p* = 0.15 in both LSCO and Nd-LSCO, whose structure in that part of the phase diagram is orthorhombic, and T* = 115 ± 10 K at *p* = 0.16 in Eu-LSCO, whose structure in that part of the phase diagram is tetragonal (LTT) (Supplementary Fig. 1). In Nd-LSCO and Eu-LSCO, T* decreases linearly all the way from *p* ~0.08 to *p* ~0.23 (blue line in Fig. 1a). On its trajectory, the T* line goes unperturbed through the LTT transition at *p* ~0.14 for Eu-LSCO (short green line) and at *p* ~0.19 for Nd-LSCO (short red line). Clearly, the pseudogap mechanism does not care about the crystal structure (Note that it is also robust against disorder^7^).

The critical doping p* at which the pseudogap phase comes to an end, however, is material-specific. In LSCO, the linear decrease in T* vs *p* comes to an end at p* = 0.18 ± 0.01^8,9^. In Nd-LSCO, resistivity measurements^4,5^ at *p* = 0.20 and above show that the T* line only comes to an end at p* = 0.23 ± 0.01. Why does T* not continue to track the dashed blue line in Fig. 1 beyond *p* = 0.17 for LSCO, or beyond *p* = 0.23 for Nd-LSCO? In Fig. 1e, we plot p* for the three single-layer cuprates LSCO, Nd-LSCO and Bi2201^10^, as a function of *p*_FS_, the doping at which the Fermi surface undergoes a change from hole-like to electron-like as determined by ARPES measurements^2,3,11,12^. Within error bars, we observe that p* = *p*_FS_, in other words, it appears that what limits p* is the constraint that the pseudogap cannot open on an electron-like Fermi surface.

In the following, we examine whether this connection is accidental or not, by independently probing how p* and *p*_FS_ evolve under the effect of hydrostatic pressure in Nd-LSCO. With a maximal *T*_c_ of only 20 K, this cuprate provides a window into the pseudogap phase near its end point at p*, free of superconductivity, down to the *T* = 0 limit, achieved by applying a magnetic field of 30 T (or greater), whose sole effect is to suppress superconductivity and reveal the underlying normal state (ref. ^5^). Our measurements reveal a large and unexpected downward shift of p* with pressure, which we find to be driven by a corresponding change in *p*_FS_, so that the relation p* ≤ *p*_FS_ is obeyed. This fundamental property has direct and fundamental implications for the mechanism of pseudogap formation.

## Results

### Determination of p* and *p*_FS_

**Fig. 2:**
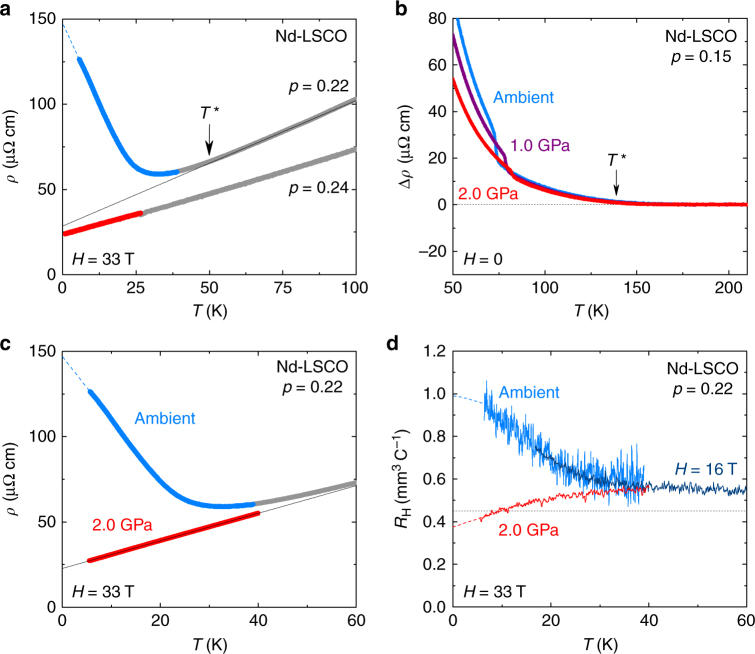
Effect of pressure on Nd-LSCO. Transport properties of Nd-LSCO as a function of temperature, at different dopings as indicated. In **a**, **c** and **d**, grey data are in zero field and coloured data are in magnetic field. **a** Resistivity of Nd-LSCO at *p* = 0.22 from ref. ^5^ (blue) and at *p* = 0.24 from ref. 4 (red), in *H* = 33 T and ambient pressure. At *p* = 0.22, *ρ*(*T*) is seen to deviate upwards from its linear *T* dependence at high *T* (black line) below the onset temperature T* of the pseudogap phase (Fig. 1a). **b** Difference between the data and the linear fits of Supplementary Fig. 6, showing that the onset of the upturn at T* (arrow) is independent of pressure. The sharp kink is due to the structural transition from orthorhombic at high *T* (LTO) to tetragonal at low *T* (LTT) (see Fig. 1). **c** Resistivity of Nd-LSCO at *p* = 0.22 and *H* = 33 T, at ambient pressure (blue, ref. 5) and *P* = 2.0 GPa (red, this work), with a linear fit (black line) to the *P* = 2.0 GPa data. **d** As in **c**, but for the Hall coefficient *R*_H_(*T*). The horizontal dotted line is the value expected from the large hole-like Fermi surface. Coloured dashed lines are a guide to the eye

Our study is based on transport signatures of p* and *p*_FS_. We begin with p*. When *p* < p*, the electrical resistivity *ρ*(*T*), Nernst coefficient and Hall coefficient *R*_H_(*T*) all exhibit large upturns at low temperature^4,5,6,9^—signatures of the pseudogap phase, attributed to a drop in carrier density *n* from *n = *1 + *p* above p* to *n = p* below p*^5,9,13^. In Nd-LSCO at *p* = 0.20, ARPES sees a gap opening at T* = 75 K (ref. 3), precisely the temperature below which the resistivity exhibits an upward deviation from its high-*T* linear behaviour^4,5^. By contrast, at *p* = 0.24 the three transport coefficients show no trace of any upturn at low *T* ^4,5,6,^, with the resistivity remaining linear down to *T* → 0 (Fig. 2a), consistent with the absence of a gap in ARPES data^3^.

**Fig. 3:**
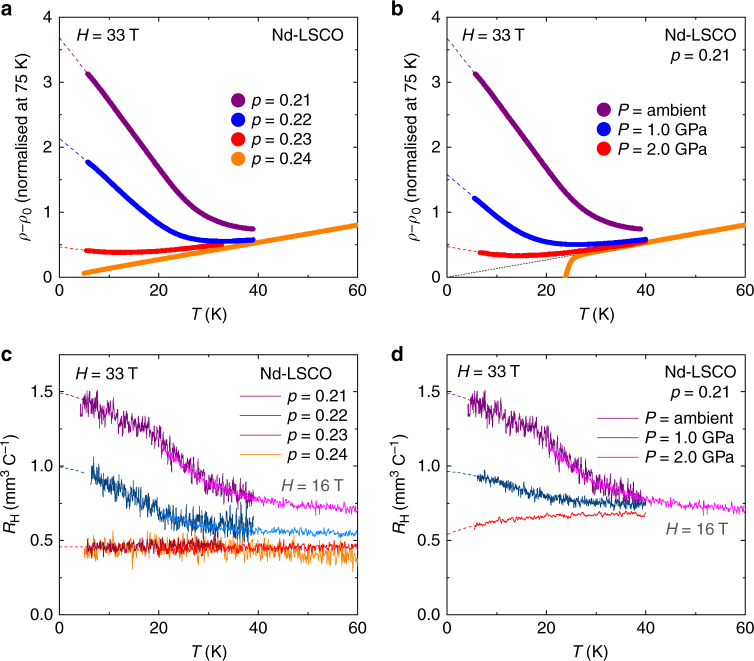
Effect of doping and pressure on Nd-LSCO. Comparing the effect of doping on the pseudogap phase in Nd-LSCO (left panels) to the effect of pressure (right panels). **a**,** b** Electrical resistivity expressed as Δ*ρ* = *ρ*(*T*) − *ρ*_0_ (normalised at 75 K), where *ρ*_0_ is the residual resistivity estimated from a linear fit to *ρ*(*T*) above T* (ref. ^5^). The values of Δ*ρ* at *T* → 0 are plotted vs *p* in Fig. 4a. **a** At ambient pressure, for various dopings as indicated, in *H* = 33 T (data from ref. ^5^). **b** At *p* = 0.21, for different pressures as indicated, in *H* = 33 T. Data at *P* = 2.0 GPa and *H* = 0 are also shown (orange), together with a linear extrapolation to *T* = 0 (black dotted line). **c**,** d** Same as in **a** and **b** but for the Hall coefficient *R*_H_. Light and dark coloured curves are in *H* = 16 and 33 T, respectively. The values at *T* → 0, labelled *R*_H_(0), are plotted vs *p* in Fig. 4b. All dashed lines are a guide to the eye

**Fig. 4:**
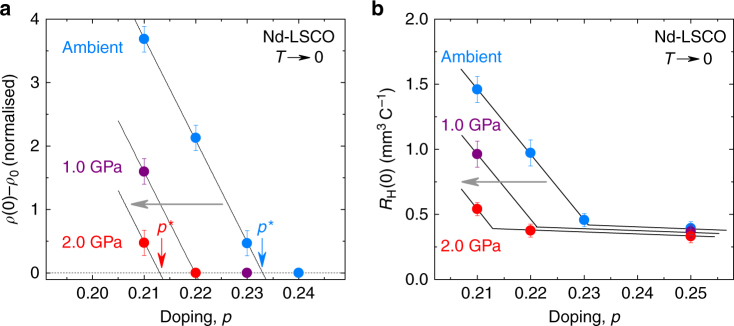
Effect of pressure on p*. **a** Magnitude of the upturn in *ρ*(*T*) at *T* → 0 and for *H* = 33 T as a function of doping, for ambient pressure (blue), *P* = 1.0 (purple) and 2.0 GPa (red), obtained from ambient pressure data (Fig. 3a) and from pressure data at *p* = 0.21 (Fig. 3b), *p* = 0.22 (Fig. 2c) and *p* = 0.23 (Supplementary Fig. 7). The pseudogap critical point p* is where the upturn goes to zero (arrow). **b** *R*_H_(0) vs doping for ambient pressure (blue), *P* = 1.0 (purple) and 2.0 GPa (red), obtained from ambient pressure data at *p* = 0.21, 0.22 and 0.23 (Fig. 3c) for *H* = 33 T, and *p* = 0.25 (Supplementary Fig. 4) for *H* = 16 T, and from pressure data at *p* = 0.21 (Fig. 3d) and *p* = 0.22 (Fig. 2d) for *H* = 33 T, and *p* = 0.25 (Supplementary Fig. 4) at *H* = 16 T. All lines are a guide to the eye. The effect of pressure is to shift p* down in doping (grey arrows), by roughly dp* = −0.02 for 2.0 GPa. All the error bars reflect the uncertainty on the extrapolation to *T* = 0

In Fig. 3, we reproduce published data^5^ for *ρ*(*T*) (Fig. 3a) and *R*_H_(*T*) (Fig. 3c) in Nd-LSCO at different dopings. The upturns decrease as *p* approaches p* from below. We determine p* as the doping where the upturns in *ρ*(*T*) and *R*_H_(*T*) vanish, giving p* = 0.23 ± 0.01 (Fig. 4). (Note that at that doping *ρ*(*T*) displays a slight upturn while *R*_H_(*T*) remains flat. This difference comes possibly from the fact that *ρ*(*T*) is sensitive to the total carrier density while *R*_H_(*T*) is balanced by electron- and hole-like contributions, as in the reconstructed Fermi surface just below p* for an anti ferromagnetic scenario^14^. See discussion in ref. ^5^).

We now turn to *p*_FS_. In a single-layer cuprate, the Fermi surface changes topology from hole-like at *p* < *p*_FS_ (Fig. 1c) to electron-like at *p* > *p*_FS_ (Fig. 1d). (*p*_FS_ is the doping at which the van Hove singularity in the density of states crosses the Fermi level.) ARPES studies show that the Fermi surface is hole-like in LSCO at *p* = 0.17^11^ and Nd-LSCO at *p* = 0.20^3^, while it is electron-like at *p* = 0.20^2^ and *p* = 0.24^3^, respectively, so that *p*_FS_ = 0.19 ± 0.01 in LSCO and *p*_FS_ = 0.22 ± 0.01 in Nd-LSCO.

### Changes under hydrostatic pressure

**Fig. 5:**
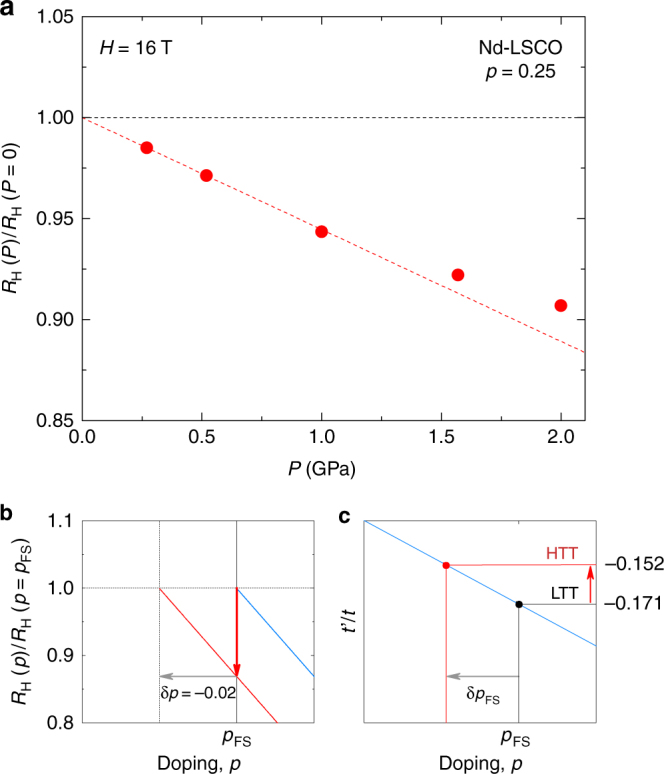
Effect of pressure on *p*_FS_. **a** Pressure dependence of the Hall coefficient in Nd-LSCO at *p* = 0.25 > p*, normalised by its value at ambient pressure, *R*_H_(0) = 0.4 mm^3^ C^−1^ (Supplementary Fig. 4). The dashed line is a linear fit through the data at low pressure, whose slope is −6 % GPa^−1^. **b** Sketch of the doping dependence of *R*_H_ in LSCO (Supplementary Fig. 3), normalised by its value at *p*_FS_ (blue line). The red line illustrates the effect of shifting *p*_FS_ down in doping by an amount δ*p*_FS_ = −0.02 (grey arrow): *R*_H_ decreases by ~13 % (red arrow). This is the amount by which *R*_H_ decreases in Nd-LSCO under 2.0 GPa, for p > p* (**a**). **c** Sketch of the theoretical dependence of *p*_FS_ on the band–structure parameter *t*’/*t*. For a given value of *t*’/*t*, *p*_FS_ is given by the blue line, which separates the regions where the cuprate Fermi surface is electron-like (above) and hole-like (below). Our band–structure calculations for Nd-LSCO yield *t*’/*t* = −0.171 in the LTT structure (black) and *t*’/*t* = −0.152 in the HTT structure (red) (Supplementary Fig. 2), so that *p*_FS_ is expected to decrease (grey arrow) under pressure, as observed experimentally

We now examine the effect of pressure on *p*_FS_ and p*. Pressure is known to change the crystal structure of Nd-LSCO from LTT at ambient pressure to HTT at *P* > 4.2 GPa^15^. Our band–structure calculations based on a standard tight-binding model (see 'Methods' section and Supplementary Fig. 2) show that this causes a decrease in the ratio |*t*’/*t*|, where *t* and *t*’ are nearest- and next-nearest-neighbour hopping parameters. It is a property of this model that the Fermi surface goes from hole-like to electron-like with decreasing |*t*’/*t*| (at fixed*p*), consistent with fits to ARPES data on LSCO^2,11^. Pressure applied to Nd-LSCO is therefore expected to reduce *p*_FS_ (Fig. 5c). Although the maximum pressure in our experiment is 2.0 GPa, it still reduces the CuO_6_ octahedron tilt angle of the LTT structure significantly towards the HTT phase^15^, which decreases |*t*’/*t*|.

Experimentally, we study the pressure dependence of *p*_FS_ by looking at *R*_H_ for *p* ≥ p*. In LSCO in the regime above *p*_FS_ ~0.19, *R*_H_ decreases linearly with doping as the system moves away from *p*_FS_ (Supplementary Fig. 3), to eventually become negative above*p*~0.35^16,17^. Quantitatively, an increase in doping by δ*p* = 0.02 corresponds to a ~13% drop in *R*_H_ (Fig. 5b). Since all there is in this regime is a single large electron-like Fermi surface, Nd-LSCO in the regime above *p*_FS_ ~0.22 must display a similar linear decrease of *R*_H_ with doping, possibly with a different absolute value. Hence, the same relative change in *R*_H_ is expected to correspond to a similar displacement in doping. In Fig. 5a (see also Supplementary Fig. 4), we see that *R*_H_ in Nd-LSCO at *p* = 0.25 decreases by ~12% under 2 GPa, which implies that *p*_FS_ in Nd-LSCO shifts down by δ*p*_FS_ ~0.02 under 2 GPa, based on the LSCO data.

To study the pressure dependence of p*, we measured the high-field normal-state resistivity under pressure up to 2 GPa. Our data on LSCO below p* show that the pseudogap is unaffected by pressure, in the sense that the amplitude of the low-*T* upturn in the resistivity at *p* = 0.143 (ref. ^9^) is unchanged (Supplementary Fig. 5). In Nd-LSCO at *p* = 0.15, our measurements (Fig. 2b and Supplementary Fig. 6) also show that T* is independent of pressure, as previously observed in YBa_2_Cu_3_O_*y*_ (ref. ^18^). So a pressure of 2 GPa does not tune T* directly. Nevertheless, in Nd-LSCO just below p* we observe a dramatic effect of pressure on the pseudogap: at *p* = 0.22, 2.0 GPa completely eliminates the low-*T* upturn in the resistivity (Fig. 2c), resulting in a linear *T* behaviour characteristic of the regime at p* and above (refs. ^4,8^). A suppression of the low-*T* upturn is also seen at *p = *0.21 (Fig. 3b). This large and unexpected effect of pressure on the pseudogap is our main experimental finding.

In Fig. 3, we compare the effect of pressure on the resistivity and Hall coefficient of Nd-LSCO with the effect of doping. (Note that unlike for YBa_2_Cu_3_O_y_, pressure does not change the doping in Nd-LSCO, which is set by the Sr content.) We see that pressure has the same effect as doping, consistent with a lowering of p* induced by pressure. Quantitatively, p* moves down from 0.23 at *P* = 0 to 0.21 at *P* = 2.0 GPa (Fig. 4). A downward shift δp* = −0.02 in 2.0 GPa is the same shift (δ*p*_FS_ ~ −0.02) that is observed for *p*_FS_. We infer that it is the downward movement of *p*_FS_ that constrains p* to move down with pressure, thereby showing that the condition p* ≤ *p*_FS_ must hold.

## Discussion

This elucidates why the pseudogap phase of LSCO ends at a lower doping than in Nd-LSCO, for indeed p* = *p*_FS_ in LSCO, within error bars (Fig. 1e). In most cuprates, *p*_FS_ is much higher, as in the single-layer material Bi2201, for example, where *p*_FS_ = 0.41 ± 0.02 (ref. ^12^). Remarkably, p* is nearly as high, with p* = 0.38 ± 0.02 in Bi2201 (ref. ^10^), as illustrated in Fig. 1e.

Our finding that p* ≤ *p*_FS_ is consistent with the known properties of all cuprates. In particular, it holds true for all known single-layer cuprates, including not only those in Fig. 1e, but also Tl_2_Ba_2_CuO_6+δ_, for example, where at *p* ~0.3 the Fermi surface is hole-like and there is no pseudogap^19^. It also holds for bi-layer cuprates, such as Bi_2_Sr_2_CaCu_2_O_8+δ_, in the sense that the pseudogap opens only once^20^ or after^21^ both Fermi surfaces (bonding and anti-bonding) have become hole-like^22^. The close proximity of p* to a van Hove singularity (at *p*_FS_) may have some impact on the physics of the pseudogap. However, since the velocity vanishes at the van Hove point, we do not expect any singularity in the transport properties. Moreover, in the presence of substantial *c*-axis dispersion, of the order of *t*_*z*_ ~20 meV in Nd-LSCO, the divergence in the density of states gets cut off at low temperature^23^.

It is striking that a minute change in the Fermi surface, smaller than that illustrated between Fig. 1c (*p* slightly below *p*_FS_) and Fig. 1d (*p* slightly above *p*_FS_), can switch off a gap of magnitude ~20 meV (ref. ^3^). This extreme sensitivity of the pseudogap on the details of the Fermi surface suggests that for the pseudogap to form it is necessary that the Fermi surface intersects the anti ferromagnetic zone boundary (AFZB), the dashed line in Fig. 1d, as proposed in refs. ^20,24^. This is precisely what happens when the doping drops below *p*_FS_. There is empirical evidence that this intersection may indeed be a crucial element of the pseudogap mechanism. First, this AFZB is where in *k*-space the separation of ungapped and (pseudo)gapped states occurs, as detected by quasiparticle interference in scanning tunnelling microscopy^21^. In other words, the AFZB is the pseudogap phase’s organising principle in *k*-space: it defines the so-called 'Fermi arcs'. Note thatwhen the pseudogap turns on (with decreasing *T* or *p*), the *k*-space area contained by the ungapped states (between the Fermi arcs and the AFZB) goes from *A* ~ 1 + *p* to *A* ~ *p* (ref. ^21^). Second, upon crossing p* from above, the carrier density measured by the Hall number goes from *n* = 1 + *p* to *n* = *p* (refs. ^5,13^), consistent with *A* ~ *n*. The simplest way to obtain a loss of 1.0 hole per planar Cu atom is to reconstruct the large hole-like Fermi surface (with 1 + *p* holes) by folding it about the AFZB, which produces four small nodal hole pockets (with *p* holes)^14^. This can be achieved either by AF order with a wavevector **Q** = (*π*, *π*) or by an Umklapp surface coincident with the AFZB, as in the YRZ model^25^. If this is indeed how the pseudogap phase transforms the Fermi surface, then no Fermi arcs (or nodal pockets) can form when *p* > *p*_FS_. Electron-doped cuprates provide a clear example of Fermi surface reconstruction caused by long-range AF order, where the AFZB plays a key role, but where no pseudogap phase forms^26^. The issue of why there is a pseudogap in hole-doped cuprates and not in electron-doped cuprates, however, is an open question.

If the pseudogap phase needs states near (*π*, 0) to form, the question is at what energy do those states have to be relative to *ε*_F_? We presume that they should be within the pseudogap energy Δ_PG_ of *ε*_F_. This implies that for *p* just above *p*_FS_, the pseudogap phase can still form. This introduces some 'width' to the criterion p* ≤ *p*_FS_. In a similar vein, a 3D dispersion of the Fermi surface in the *k*_z_ (or *c*) direction will also give some 'width' to the criterion, since at a given doping the Fermi surface can be electron-like at some *k*_z_ value but still hole-like at some other *k*_z_ value.

The requirement that a pseudogap cannot form in a cuprate with an electron-like Fermi surface imposes a stringent constraint on theories of the pseudogap phase^25,27–31^. In the YRZ model^25^, a pseudogap forms because carriers undergo Umklapp scattering, inherited from the Mott insulator, at points where the Fermi surface intersects the AFZB; this model therefore agrees with our proposed constraint. Our findings are also broadly consistent with spin-fermion models that hinge on the hot spots that lie at the intersection of Fermi surface and AFZB^27,28^. More specifically, two recent theoretical studies^29,32^ find that a pseudogap only opens on hole-like Fermi surfaces and that p* ≤ *p*_FS_ for a wide range of band–structure parameters, even in the strong-coupling regime where the anti ferromagnetic correlations responsible for the pseudogap are short ranged. On the other hand, in scenarios characterised by a wavevector **Q** = (0, 0), the AFZB plays no special role and there is then no obvious reason for the constraint to be effective. This would seem to rule out nematic order^31^ and intra-unit cell magnetic order^30,33^ as possible drivers of the pseudogap phase. Instead, these orders would be secondary instabilities^6^.

On the experimental side, the ability to continuously suppress and restore the pseudogap with pressure in a given sample without changing the doping or disorder level provides a promising avenue to study the pseudogap state, compatible with a range of probes such as Raman, optics, X-ray and neutron scattering.

## Methods

### LSCO samples

Single crystals of La_2−*x*_Sr_*x*_CuO_4_ (LSCO) were grown by the flux-zone technique, with nominal Sr concentrations of *x* = 0.145 at Hokkaido University and *x* = 0.18 at the University of Tokyo. Samples for resistivity measurements were cut in the shape of small rectangular platelets, of typical dimensions 1 mm × 2 mm × 0.5 mm, with the smallest dimension along the *c* axis. Contacts were made using H20E silver epoxy, diffused by annealing. The hole concentration (doping) *p* for our nominal *x* = 0.145 sample was determined using the doping dependence of the (tetragonal to orthorhombic) structural transition temperature, *T*_LTO_, which is detected in the resistivity as a small but sharp kink. This yields *p* = 0.143 (ref. ^9^). For our sample with *x = *0.18, we take *p = x*.

### Nd-LSCO samples

Single crystals of La_2−*y*−*x*_Nd_*y*_Sr_*x*_CuO_4_ (Nd-LSCO) were grown at the University of Texas at Austin with a Nd content *y* = 0.4, using a travelling-float-zone technique, and cut from boules with nominal Sr concentrations *x* = 0.15, 0.21, 0.22, 0.23 and 0.25. The samples were prepared for transport measurements as described above for the LSCO samples. For all five samples, the hole concentration *p* is given by *p* = *x*, with an error bar ±0.003. The samples labelled here *p* = 0.21, 0.22, 0.23 and 0.24 are the same samples as those studied in ref. ^5^; the sample labelled here *p* = 0.25 is a new sample, with *p* = 0.25 ± 0.003.

### Resistivity and Hall measurements

The electrical transport measurements were performed via a standard four-point low AC technique using an SR830 lock-in amplifier and a Keithley 6221 current source. A current of typically 2 mA was applied within the CuO_2_ planes and the magnetic field along the *c*-axis. The longitudinal and Hall resistances *R*_*xx*_ and *R*_*xy*_ were measured at Sherbrooke in steady fields up to 16 T and at the NHMFL in steady fields up to 45 T. The Hall resistance *R*_*xy*_ is obtained by reversing the field and anti-symmetrizing the data, as *R*_*xy*_(*H*) = (*R*_*xy*_( + *H*) − *R*_*xy*_(−*H*))/2.

### Application of pressure

Pressure was applied on our samples using a miniature non-magnetic piston-cylinder cell. The pressure medium is Daphne oil 7474, which remains liquid at all pressures measured here at 300 K, ensuring a high degree of hydrostaticity. The internal pressure is measured both at room temperature and at 4.2 K, using either the fluorescence of a small ruby chip or a Sn manometer. The values quoted throughout are the low temperature pressures. The error bar on all the pressure values is ± 0.05 GPa, which comes from the uncertainty in measuring the position of the fluorescence peaks. For each measurement, the cell was cooled slowly (<1 K min^−1^) to ensure a homogeneous freezing of the pressure medium.

### Band–structure calculations

Band–structure calculations were performed by using the full potential augmented plane wave band method, implemented in the WIEN2k package. We used the local density approximation for the exchange-correlation potential. We used 1000 k-points inside the first Brillouin zone. The convergence of total energy with respect to the number of k-points was checked to have a precision better than 0.007 eV per formula unit. We calculated the electronic structure of La_2_CuO_4_ using the structural parameters of Nd-LSCO measured experimentally by X-ray diffraction for both ambient pressure (LTT structure; ref. ^34^ and *P* = 4.2 GPa (HTT structure; ref. ^15^). In the case of *P* = 4.2 GPa (HTT), in order to determine the internal position of atoms inside the CuO_6_ octahedron, we assumed an isotropic contraction of the CuO_6_ octahedra from hydrostatic pressure. Experiments on La_2_CuO_4_ have shown this assumption to be valid^35^. Tight-binding hopping parameters were obtained by fitting Cu(d_*x*2-*y*2_)-driven bands for high-symmetry points of the anti ferromagnetic zone boundary.

### Sample size

No statistical methods were used to predetermine sample size.

### Data availability

All relevant data are available from the authors.

## Electronic supplementary material

Supplementary Information
