# Supplementary material for: Pseudogap phase of cuprate superconductors confined by Fermi surface topology
Source: Nat Commun. 2017 Dec 11;8:2044. doi: 10.1038/s41467-017-02122-x (PMC5725553; doi:10.1038/s41467-017-02122-x)
Supplement: Supplementary file 1 — Supplementary Information [file 41467_2017_2122_MOESM1_ESM.pdf]

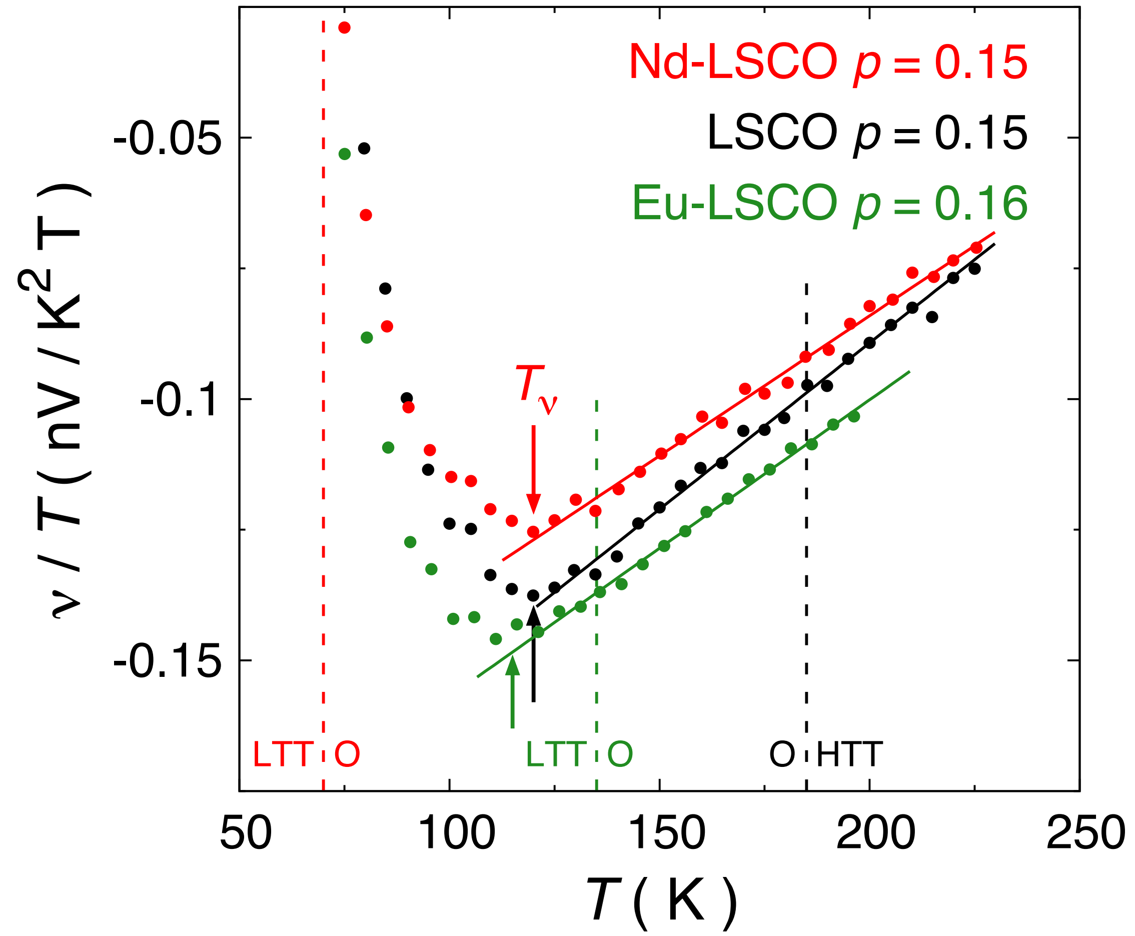

Supplementary Figure 1 | Nernst coefficient for LSCO cuprates.

Nernst coefficient ν vs temperature for LSCO p = 0.15 (black), Nd-LSCO p = 0.15 (red), and Eu-LSCO p = 0.16 (green), plotted as ν / T (figure adapted from ref. 1). The full lines are linear fits to the data and the arrows indicate the deviation from this linear behaviour at a temperature labeled T_ν . The vertical dashed lines indicate the high temperature tetragonal (HTT) to orthorhombic (O) structural transition in LSCO (black), and the orthorhombic to low temperature tetragonal (LTT) transitions in Nd-LSCO (red) and Eu-LSCO (green). T_ν is essentially the same for all three materials, independent of where the structural transitions occur.

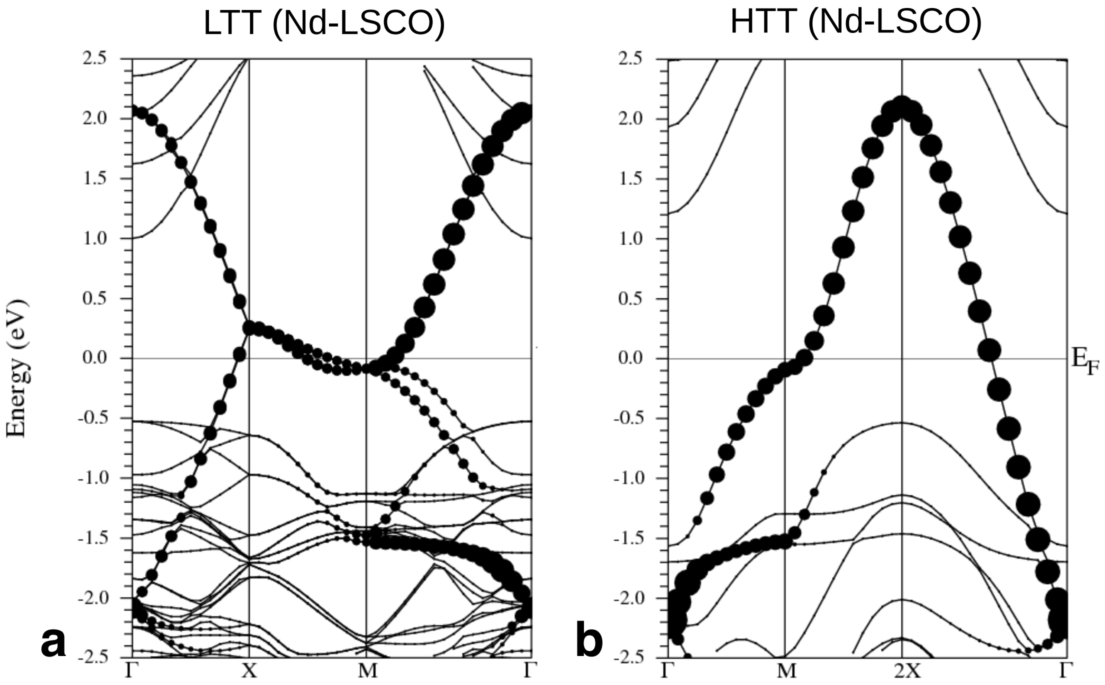

Supplementary Figure 2 | Band structure calculations.

Band-structure calculations for Nd-LSCO in the LTT structure at ambient pressure (**a**) and in the HTT structure at *P* = 4.2 GPa (**b**). The lattice parameters for the calculations are taken directly from X-ray diffraction data: ref. 2 for (a) and ref. 3 for (b). A tight-binding fit to the band structure gives the following values for the parameters *t* and *t'* : *t* = 0.520 eV, *t'* = - 0.089, and *t'* / *t* = - 0.171, for LTT (*P* = 0) ; *t* = 0.539 eV, *t'* = - 0.082, and *t'* / *t* = - 0.152, for HTT (*P* = 4.2 GPa).

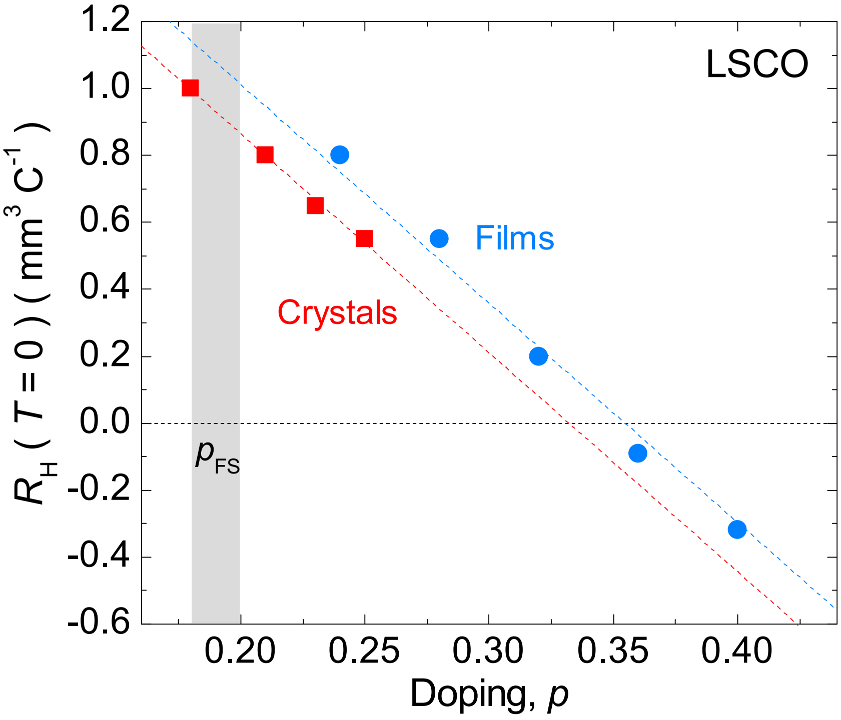

Supplementary Figure 3 | Hall coefficient vs doping in LSCO.

Hall coefficient R_H of LSCO in the T = 0 limit, as a function of doping p, for p = 0.18 and higher. Data are based on measurements made on crystals (red squares from ref. 4) and thin films (blue dots, from ref. 5). Lines are linear fits through the respective data. R_H is seen to decrease linearly as the doping increases away from p_{FS} (grey band). We use this linearity of R_H as a function of p to quantify the shift of p_{FS} with pressure in Nd-LSCO, in Figs. 4 and 5.

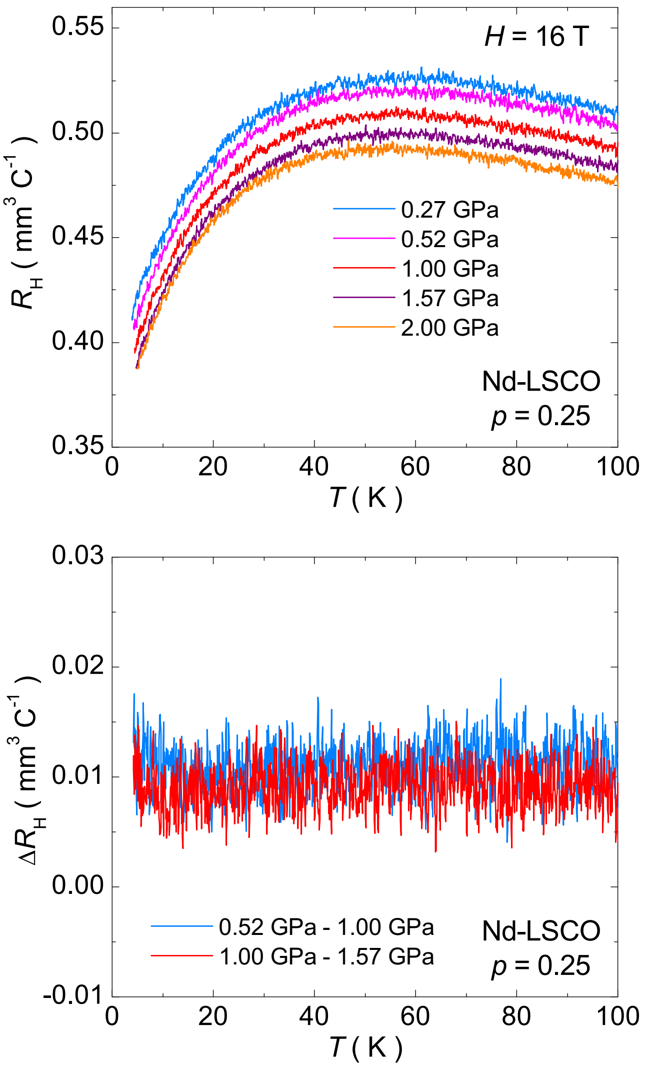

Supplementary Figure 4 | Effect of pressure on the Hall coefficient of Nd-LSCO at *p* > *p.**

a) Hall coefficient R_H of Nd-LSCO at p = 0.25 (above p^* = 0.23) as a function of temperature, measured at H = 16 T, for various applied pressures as indicated. **b)** Difference between two adjacent isobars in (**a**), for pairs as indicated. $R_H(T)$ decreases rigidly, at a rate of - 6 % GPa^{-1}, relative to $R_H(0)$ = 0.4 mm^3 C^{-1}.

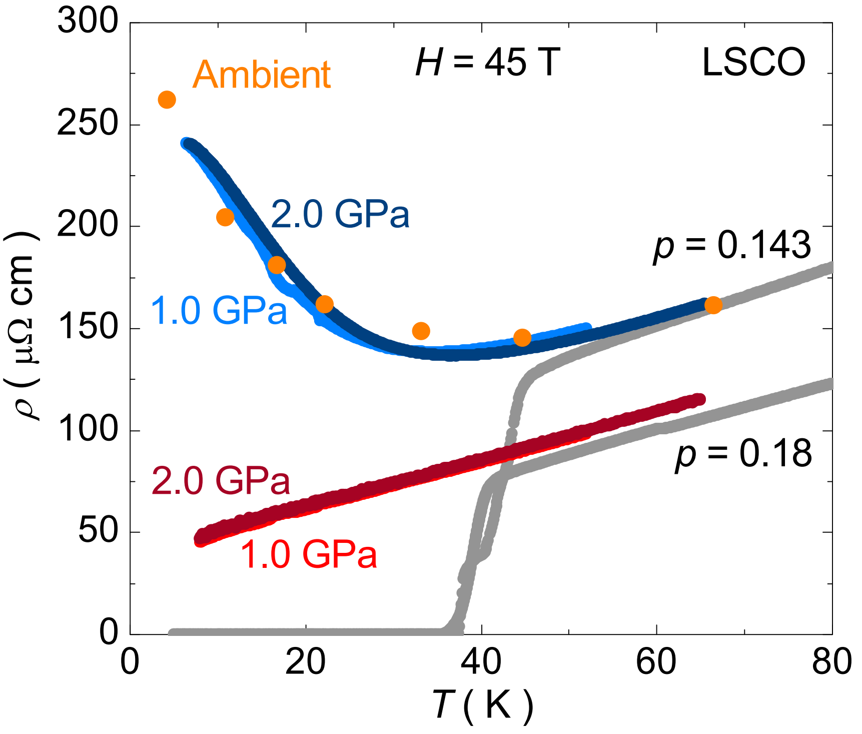

Supplementary Figure 5 | Resistivity of LSCO under pressure.

Resistivity of LSCO as a function of temperature, in H = 45 T, for p = 0.143 (blue and dark blue) and p = 0.18 (red and burgundy), at pressures as indicated. Ambient pressure data for the same dopings, from ref. 6, are also shown, in grey for H = 0 and orange for H = 55 T.

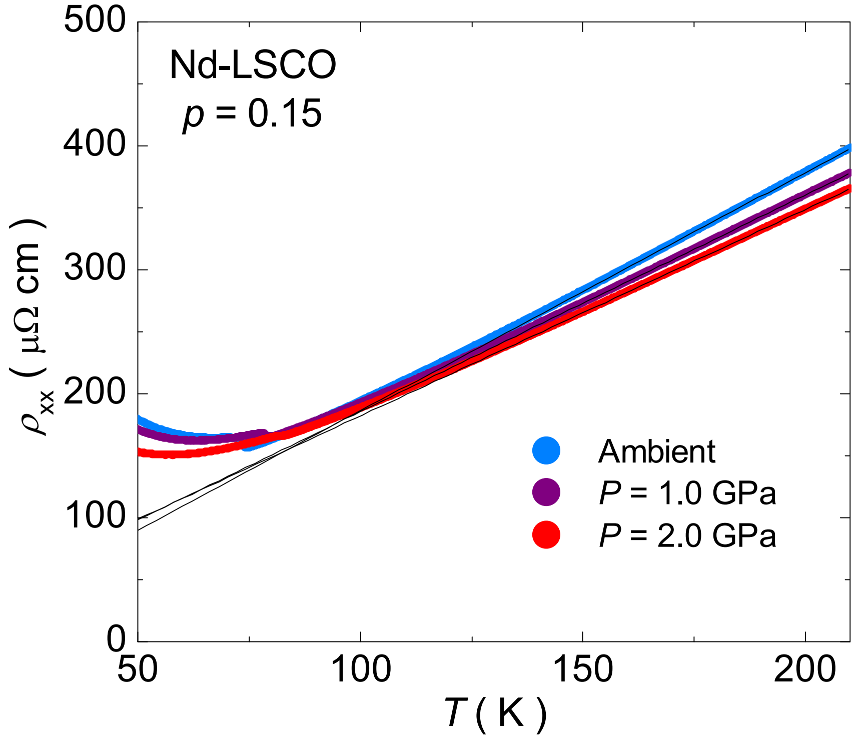

Supplementary Figure 6 | Resistivity of Nd-LSCO at *p* = 0.15.

Resistivity of Nd-LSCO as a function of temperature, at p = 0.15 and in zero magnetic field, for three different pressures as indicated. Lines are linear fits at high temperature. The difference between data and fit is plotted in Fig. 2b.

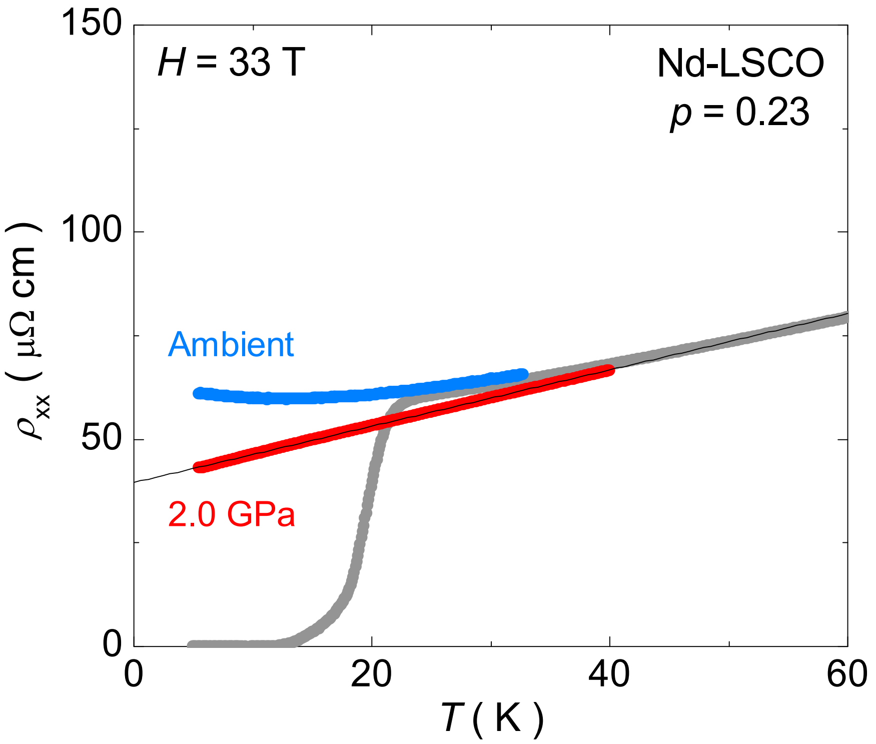

Supplementary Figure 7 | Resistivity of Nd-LSCO at *p* = 0.23.

Electrical resistivity of Nd-LSCO at $p = 0.23$ as a function of temperature, under ambient pressure (blue) and 2.0 GPa (red), in $H = 33$ T. Grey data are ambient pressure and zero field data on the same sample. The black line is a linear fit to the 2.0 GPa data.

[1] Cyr-Choinière, O. *et al*. Pseudogap temperature *T** of cuprate superconductors from the Nernst effect. Preprint at http://lanl.arxiv.org/abs/1703.06927 (2017).

[2] Axe, J. D. & Crawford, M. K. Structural instabilities in lanthanum cuprate superconductors. *J. Low Temp. Phys.* **95**, 271-284 (1994).

[3] Crawford, M. K. *et al*. High-pressure study of structural phase transitions and superconductivity in $La_{1.48}Nd_{0.4}Sr_{0.12}CuO_4$. *Phys. Rev. B* **71**, 104513 (2005).

[4] Ando, Y. *et al*. Evolution of the Hall coefficient and the peculiar electronic structure of the cuprate superconductors. *Phys. Rev. Lett.* **92**, 197001 (2004).

[5] Tsukada, I. & Ono, S. Hall coefficients of heavily overdoped $La_{2-x}Sr_xCuO_4$. *Phys. Rev. B* **74**, 134508 (2006).

[6] Laliberté, F. *et al.* Origin of the metal-to-insulator crossover in cuprate superconductors. Preprint at http://lanl.arxiv.org/abs/1606.04491 (2016).
